# Myelodysplastic syndrome/myeloproliferative neoplasm with ringed sideroblasts and thrombocytosis

**DOI:** 10.1002/jha2.299

**Published:** 2021-10-20

**Authors:** Azka Tasleem, Janet Roepke, Salahuddin Siddiqui

**Affiliations:** ^1^ Department of Internal Medicine Indiana University Health Ball Memorial Hospital Muncie Indiana USA; ^2^ Department of Pathology Indiana University Health Ball Memorial Hospital Muncie Indiana USA; ^3^ Department of Hematology/Oncology Indiana University Health Ball Memorial Hospital Muncie Indiana USA

A 72‐year‐old female presented for evaluation for almost a 5‐year history of gradually worsening anemia with hemoglobin ranging from 8 to 9 g/dl (reference range:12–15 g/dl) and thrombocytosis with platelet count ranging from 500 to 600 × 10^9^/L (reference range: 150–450 × 10^9^/L). Her white blood cell count and differential were in normal range. Laboratory evaluation revealed normal iron, vitamin B12, folate, copper, lactate dehydrogenase, haptoglobin, and reticulocyte count. Serum erythropoietin level was 55.5 mIU/ml (reference range: 2.6–18.5 mIU/ml). Complete metabolic profile was unremarkable. Physical examination showed no palpable splenomegaly. Peripheral blood film showed macrocytic anemia with anisopoikilocytosis, basophilic stippling, rare Pappenheimer bodies, and rare circulating nucleated RBCs. She underwent bone marrow biopsy with cytogenetics and next‐generation sequencing (NGS) for common myeloid mutations. Bone marrow biopsy (Figure 1) showed hypercellular marrow, megakaryocytic hyperplasia with some giant hyperlobated megakaryocytes, erythroid hyperplasia with dyserythropoiesis, and increased reticuloendothelial iron stores with >15% numerous ring sideroblasts. Myeloblasts were <1%. Cytogenetics and fluorescence in situ hybridization (FISH) testing for common myelodysplastic syndrome/myeloproliferative neoplasm (MDS/MPN) panel were normal. Patient tested negative for *BCR‐ABL1, CALR* and *MPL* mutation and NGS showed *JAK‐2* and *SF3B1* mutation.

**FIGURE 1:**
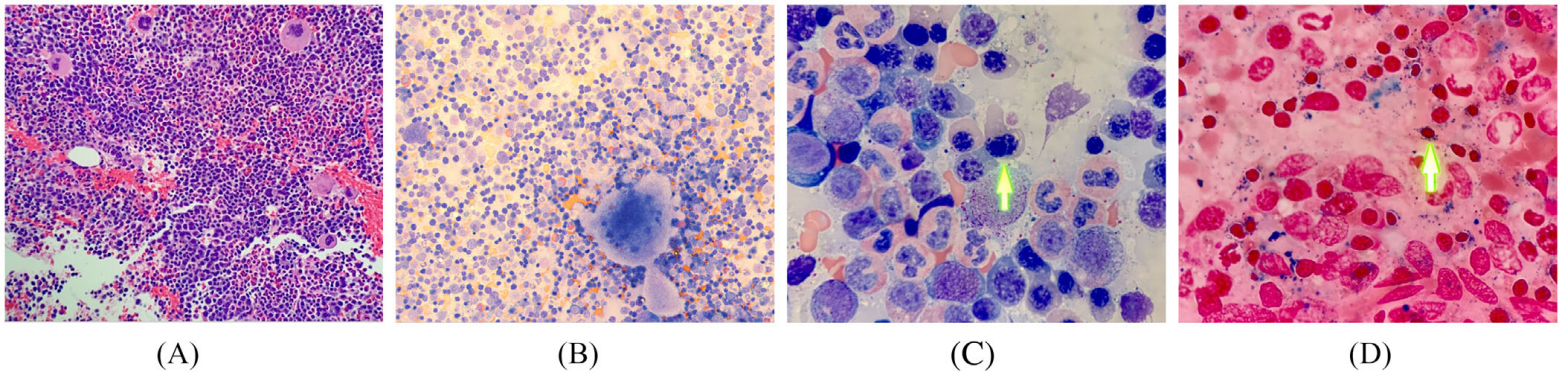
(A–D) Bone marrow biopsy (Figure 1). Hematoxylin and Eosin‐stained marrow clot section showing hypercellular marrow, trilineage hyperplasia (inset A, 20×). Bone marrow aspirate smear, stained with Wright stain showing megakaryocytic hyperplasia with an abnormal hyperlobated/hypersegmented megakaryocyte (inset B, 20×). Hematoxylin and Eosin‐stained biopsy section showing nucleated red cell precursor with irregular nuclear contours (inset C, 100× oil). Iron‐stained aspirate smear, arrow immediately below one of the ringed sideroblasts (inset D, 100× oil)

The presence of platelet count >450 × 10^9^/L, anemia, hypercellular marrow, dyserythropoiesis, and numerous ring sideroblasts led to diagnosis of myelodsyplastic/myeloproliferative neoplasm with ring sideroblasts and thrombocytosis as per 2016 WHO Classification [1]. With thrombocytosis, aspirin can reduce the risk of thrombosis and vasomotor symptoms [2]. In patients with prior history of arterial/venous thrombosis and age >60, the risk of thrombosis is higher [3]; hence, cytoreductive therapy with hydroxyurea can also be considered in addition to aspirin. Anemia is treated using erythropoiesis stimulating agents and transfusion support. If patient has associated anemia and is hydroxyurea‐intolerant, then agents such as lenalidomide, anagrelide, and interferon alpha are used to prevent worsening of anemia [4]. Since our patient had both thrombocytosis and anemia, she was started on anagrelide for cytoreduction, which has a lower risk of causing anemia.

## CONFLICT OF INTEREST

The authors declare no conflict of interest.

## AUTHOR CONTRIBUTIONS

Azka Tasleem and Salahuddin Siddiqui wrote the initial draft of the manuscript. Janet Roepke contributed to pathologic interpretations. The final version of the manuscript was approved by all authors.

## Data Availability

The data that support the findings of this study are available from the corresponding author upon reasonable request.
